# Antibiotic use at the Centre Hospitalier Universitaire de Zone d'Abomey Calavi/Sô-Ava (CHUZ/AS) in Benin: a point prevalence survey

**DOI:** 10.1093/jacamr/dlae220

**Published:** 2025-01-10

**Authors:** Morelle Sèssiwèdé Gnimavo, Bawa Boya, Steward Mudenda, Aurel Constant Allabi

**Affiliations:** Laboratory of Pharmacology and Toxicology, University of Abomey-Calavi, 05 BP 1604 Cotonou, Benin; Teaching Hospital of Abomey-Calavi/Sô-Ava, 05 BP 1604 Contonou, Benin; Laboratory of Biology and Molecular Typing in Microbiology (LBTMM), University of Abomey-Calavi, 05 BP 1604 Cotonou, Benin; Department of Pharmacy, School of Health Sciences, University of Zambia, Lusaka, Zambia; Surveillance and Research Technical Working Group, Antimicrobial Resistance Coordinating Committee, Zambia National Public Health Institute, Lusaka, Zambia; Laboratory of Pharmacology and Toxicology, University of Abomey-Calavi, 05 BP 1604 Cotonou, Benin; Teaching Hospital of Abomey-Calavi/Sô-Ava, 05 BP 1604 Contonou, Benin

## Abstract

**Background:**

Antimicrobial stewardship promotes the appropriate use of antibiotics to prevent the emergence and spread of antimicrobial resistance. This study evaluated the use of antibiotics using a point prevalence survey at the Centre Hospitalier Universitaire de Zone d'Abomey Calavi/Sô-Ava (CHUZ/AS) in Benin.

**Methods:**

This cross-sectional study utilized the WHO point prevalence survey methodology for monitoring antibiotic use among inpatients in hospitals. The survey was conducted from 11 January 2022 to 19 January 2022 among hospitalized patients before 8:00 a.m. on the day of the survey.

**Results:**

Of the 111 inpatient medical files reviewed, the prevalence of antibiotic use was 82.9%. The number of antibiotics received per patient ranged from 1 to 5, with a mean of 2.45 ± 1.11 and a median of 2. The most commonly prescribed class of antibiotics was beta-lactams (46.7%), aminoglycosides (20.6%) and nitroimidazoles (19.7%). According to the WHO AWaRe classification, 30.4% of inpatients received the Access group of antibiotics and 44% received a combination of Access and Watch group antibiotics; treatment was empiric in 94.5% of encounters. Only 22.7% of patients were treated based on microbiological examination/culture and sensitivity testing.

**Conclusions:**

This study found a high prevalence of antibiotic use among inpatients at the CHUZ/AS Tertiary Care Hospital in Benin. The most prescribed antibiotics were ampicillin, metronidazole and ceftriaxone. Consequently, the study found a low use of culture and sensitivity testing to guide treatment, particularly in the paediatric and surgical population, and the preference for broad-spectrum antibiotics suggests that antibiotic use at the CHUZ/AS Tertiary Care is not optimal. Therefore, antimicrobial stewardship programmes, policies and guidelines must be instigated and strengthened to address these gaps and promote rational use of antibiotics.

## Introduction

Antimicrobial resistance (AMR) threatens global public health and sustainable development, with adverse health and economic consequences, unless evidence-based efforts are implemented to control its emergence and spread.^1–3^ The health and social consequences of AMR include increased morbidity and mortality, rising healthcare costs and an anticipated negative impact on economic growth.^4^ More than 700 000 people die each year from AMR, a figure that is expected to rise to 10 million annually by 2050 if decisive action is not taken.^3,5^ A recent estimation of 39 million deaths due to AMR has been reported.^6^ Less is known about the impact of this problem in African countries although the burden is considered high.^7^

The overuse and misuse of antimicrobials are considered important drivers of AMR.^8–11^ In the USA, around 30% of antimicrobial treatments are considered inappropriate.^12^ In African countries, these estimates exceed 45% and vary within primary and tertiary care settings.^13,14^ Thus, healthcare facilities where antimicrobials are frequently used are high-risk environments for the selection and spread of resistant bacteria.^15^ There can be significant variation in antibiotic use between facilities,^16^ with greater variations observed between facilities experiencing different rates of nosocomial infections and serving populations with varying burdens of disease. In low- and middle-income countries (LMICs), the burden of AMR is likely to be high and access to antibiotics is largely unregulated.^3,17–20^ Data of this nature are particularly important for countries in the process of developing and implementing national action plans against AMR.^21^

Antimicrobial stewardship (AMS) programmes to optimize antibiotic use have been successfully implemented in high-income countries without increasing healthcare costs.^22–25^ Evidence has indicated that AMS programmes promote rational use of antibiotics and improve patient outcomes.^26–28^ Point prevalence surveys (PPS) are recommended to monitor the prescribing patterns of antibiotics and adherence to treatment guidelines.^29–31^ Generally, there is a high use of antibiotics, especially in the sub-Saharan African region.^32^ Some PPS have reported a high prescribing and use of antibiotics in hospitalized patients above the recommended threshold.^31,33–36^ Therefore, through PPS, hospitals can monitor their prescribing patterns and use of antibiotics in hospitalized patients and make decisions if deviations are observed.

To promote the rational use of antibiotics, the WHO developed the Access, Watch and Reserve (AWaRe) classification of antibiotics in 2017.^37–40^ The WHO had recommended that hospitals should be using at least 60% of Access antibiotics in hospitalized patients.^38,40^ The 2024 United Nations General Assembly recommended that by 2030, hospitals should be using at least 70% of Access group antibiotics which have relatively minimal side effects and lower potential to cause AMR.^41^ Access antibiotics include antibiotics that must readily be available in hospitals; they are generally narrow spectrum, have few side effects, have less potential to develop AMR and are used for empiric treatment for most common infections.^42,43^ The Watch group antibiotics include antibiotics that are usually used for sicker patients in hospital settings and have a higher potential to develop AMR, thereby must be monitored carefully during use and should not be overused or misused.^42,43^ The Reserve antibiotics include antibiotics that are reserved to treat multidrug-resistant (MDR) pathogens.^40,42,43^ By adhering to the WHO AWaRe classification of antibiotics, prescribers can use antibiotics rationally as guided.^44,45^ Consequently, some studies have reported high use of Watch group antibiotics, thereby deviating from the WHO recommendations.^46–49^ The deviations indicate the need for AMR programmes in such facilities with inappropriate use of antibiotics.

In Benin, antibiotic prescribing patterns and AMS efforts are not widely documented. In addition, high antibiotic prescribing in hospitals and dispensing without prescription in pharmacies mean that bacteria are constantly evolving towards resistance in Benin.^7,50^ Additionally, a previous study found a high prevalence of nosocomial infections and resistance rates to antibiotics.^51^ This study was conducted as part of continuous quality improvement approaches and efforts to build capacity for surveillance of antibiotic use in healthcare facilities, with a long-term goal of linking with the AMR containment programme at the Centre Hospitalier Universitaire de Zone d'Abomey Calavi/Sô-Ava (CHUZ/AS) in Benin as well as with national efforts to combat AMR. This study evaluated antibiotic use using a PPS at the CHUZ/AS in Benin.

## Materials and methods

### Study design, site and population

This cross-sectional study, utilizing a PPS approach, was conducted at the CHUZ/AS in Benin from 11 January 2022 to 19 January 2022. This survey was conducted according to the WHO protocol for PPS studies.^29^ Patients were eligible if admitted before 8:00 a.m. on the day of the survey. The hospital offers teaching and research services in the urban area of Benin. Abomey-Calavi is a commune in the Atlantique Department of Benin, with a population of more than 407 116. Patients from the following departments were not eligible: radiology; ear, nose and throat; rehabilitation; and short-stay. Newborns born and admitted before 8:00 a.m. were included and considered as individuals distinct from their mothers. For each day of the survey, a census counted the total number of patients in a selected department. Informed consent was obtained from participants or their guardians before enrolment and accessing their medical records for data abstraction.

### Sample size estimation

The CHUZ/AS has a bed capacity of 430. Based on the WHO PPS protocol, all patients who meet the inclusion criteria must be included in the survey for any hospital with a bed space of less than 500.^29^ Therefore, we did not calculate the sample size, but we purposefully selected and included all patients that met the inclusion criteria and consented to be part of the study.

### Data collection

We used versions of the WHO PPS forms to collect ward and patient-level data.^29^ Six departments (general internal medicine, paediatrics, neonatal, surgery and emergency) of this hospital were involved in the PPS. Hospital-level data were collected by the survey coordinator, who conducted face-to-face interviews with hospital administrators. Patient-level data were collected by a two-member team consisting of a clinical or medical manager and a pharmacist. The teams extracted data from patients’ medical records, treatment records and nurses’ notes. Data included patient demographics, healthcare exposures, antibiotics used, diagnoses for which current antibiotics were administered and culture and susceptibility tests performed on current admission. Where not explicitly indicated, prophylactic antibiotic use was inferred for patients undergoing surgical procedures for which prophylactic antibiotics are recommended and for HIV-infected patients receiving cotrimoxazole. Diagnoses/indications for which antibiotics were prescribed were recorded according to predefined PPS indication codes.^29^ Adherence to treatment guidelines was assessed using the local treatment guidelines as the national treatment guidelines are currently being developed. We also categorized antibiotics into the WHO AWaRe groups.^37,38,40,42,52^

### Data analysis

The collected data were entered in Microsoft Excel 2013. Data were analysed using R ver. 4.2.1. The outcome variable in this study was patients on antibiotics (yes or no). To determine the factors that influenced prescribing patterns of antibiotics for inpatients, a univariate analysis was first performed, from which all variables with *P* < 0.2 were used to build the model. Multiple logistic regression was used to determine the factors that influenced antibiotic use among inpatients. All statistical tests were conducted at a 95% CI, and a *P* < 0.05 was considered statistically significant.

### Ethical approval

The survey protocol was approved by the National Ethics Committee for Health Research (CNERS-Benin). Clearance to collect data was obtained from the hospital leadership/management. The purpose of the survey was explained to the hospital management. We observed confidentiality throughout the process of the survey. Further, only patients who provided informed consent were enrolled in the study.

## Results

### Demographic and clinical characteristics of patients at the CHUZ/AS in 2022

The survey lasted 9 days at the CHUZ/AS. A total of 111 patients were included in the study, with as many women as men (sex ratio = 0.98). More than half of the study population was under 18 years of age (Table 1).

**Table 1.** Patient demographics at the CHUZ/AS in 2022

Variable	Attribute	Absolute frequency	Percentage
Age group	0–9 days	32	28.8
	1–24 months	10	9.0
	2–18 years	17	15.3
	18–49 years	42	39.8
	≥50 years	10	9.0
Gender	Female	56	50.5
	Male	55	49.5
Services	Neonatal	32	28.8
	Maternity	28	25.2
	Paediatrics	20	18.2
	Surgical	16	14.1
	Medicine	10	9.0
	Emergency	5	5.5

This study found that 29.7% of patients had urinary catheters. All patients had a peripheral catheter, except one patient who had a central catheter. Additionally, 38.7% of patients had undergone surgery since admission (Table 2).

**Table 2.** Clinical characteristics of patients at the CHUZ/AS in 2022 before surgery

Question	Response	Absolute frequency	Percentage
Presence of peripheral catheter	Yes	111	100
	No	0	0
Presence of a central catheter	Yes	1	0.9
	No	110	99.1
Presence of urinary catheter	Yes	33	29.7
	No	78	70.3
Surgical procedures since admission	Yes	43	38.7
	No	68	61.3

### Prevalence and factors associated with antibiotic use at the CHUZ/AS in 2022

Of the 111 patients included in this study, 82.9% were on antibiotics. The prevalence of antibiotic use was over 50% in all departments except emergency.

Univariate analysis of this study showed that hospitalization department (*P* < 0.001), age (*P* < 0.004) and surgery since admission (*P* < 0.017) were associated with increased antibiotic use at the CHUZ/AS.

Multiple logistic regression had shown that the independent predictors of antibiotic use were surgery since admission (*P* < 0.001) and age (*P* < 0.001). Patients with surgery since admission were 9.8 times more likely to be on antibiotics (CI 2.38–52.7). Newborns were 34.6 times more likely to be on antibiotics than patients aged over 50 years (CI 3.96–782) (Table 3).

**Table 3.** Factors associated with antibiotic use among patients in six departments at the CHUZ/AS in 2022

Variables	Antibiotic use *n* (%)		Univariate model		Multivariate model	
	No	Yes	Odds ratio (95% CI)		No	Yes
Services				<0.001		
Emergencies	3 (15.79)	2 (2.17)	1			
Surgical	0 (0.00)	16 (17.39)	0 (—)			
Neonatal	1 (5.26)	31 (33.70)	46.5 (4.08; 1259)			
Maternity	4 (21.05)	24 (26.09)	9 (1.16; 88.2)			
Medicine	3 (15.79)	7 (7.61)	3.5 (0.39; 40.0)			
Paediatrics	8 (42.11)	12 (13.04)	2.3 (0.31; 20.2)			
Age group				0.004		<0.001
≥50 years	4 (21.05)	6 (6.52)	1		1	
0–29 days	1 (5.26)	31 (33.70)	20,7 (2.54; 445)		34.6 (3.96; 782)	
1–24 months	2 (10.53)	8 (8.70)	2.67 (0.38; 24.3)		4.47 (0.60; 44.3)	
2–18 years	7 (36.84)	10 (10.87)	0.95 (0.18; 4.69)		0.84 (0.14; 4.89)	
18–49 years	5 (26.32)	37 (40.22)	4.93 (0.99; 4.69)		1.84 (0.29; 11.1)	
Gender				0.0852		
Female	13 (68.42)	43 (46.74)	1			
Male	6 (31.58)	49 (53.26)	2.5 (0.86; −7.06)			
Presence of a central line				0.539		
No	19 (100.00)	91 (98.91)	1			
Yes	0 (0.00)	1 (1.09)	—	—		
Presence of urinary catheter				0.350		
No	15 (78.95)	63 (68.48)	1			
Yes	4 (21.05)	29 (31.52)	1.72 (0.53; 5.65)			
Surgery since admission				0.017		0.001
No	16 (84.21)	52 (56.52)	1		1	
Yes	3 (15.79)	40 (43.48)	4.10 (1.12; −15.04)		09.8 (2.38; 52.7)	

### Distribution of patients on antibiotics by indication at the CHUZ/AS in 2022

Indications for antibiotic therapy were mainly for community-acquired infections (CAIs) (38%) and prophylaxis (62%).

### Distribution of patients on antibiotics according to the number of antibiotics prescribed per patient at the CHUZ/AS in 2022

**Figure 1.**
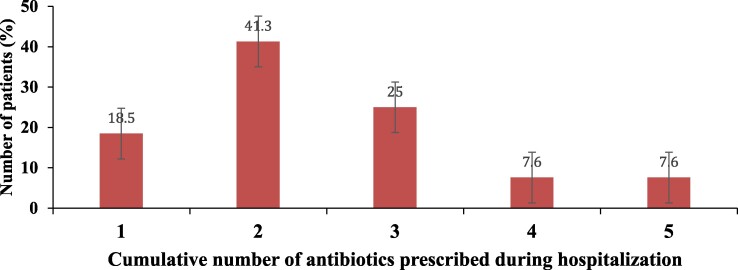
Number of antibiotics prescribed per antibiotic patient at the CHUZ/AS in 2022.

The number of antibiotics received by patients since hospitalization ranged from 1 to 5, with an average of 2.45 ± 1.11 and a median of 2. However, 15.2% of patients had received four or five antibiotics (Figure 1). This average number of antibiotic use was higher than two in all departments except emergency (Figure 1).

### Classes of antibiotics prescribed at the CHUZ/AS in 2022

This study found that penicillins, cephalosporins and aminoglycosides were the most commonly used antibiotics (˃20%) at the CHUZ/AS in 2022 (Table 4).

**Table 4.** Antibiotic families and subfamilies prescribed at the CHUZ/AS in 2022

Antibiotic families and subfamilies		Frequency	Percentage
Beta-lactam	Penicillins	56	24.9
	Cephalosporins	50	22.2
Aminoglycosides		46	20.4
Nitroimidazoles		44	19.6
Fluoroquinolones		24	10.7
Macrolides and sulfonamides		5	2.2
Total		225	100

### Types of antibiotics prescribed by indication at the CHUZ/AS in 2022

For CAIs, ampicillin and amikacin were the most prescribed antibiotics, while for surgical prophylaxis, ceftriaxone and metronidazole were the most prescribed antibiotics. For medical prophylaxis, gentamicin and ampicillin were the most prescribed antibiotics (Table 5).

**Table 5.** Antibiotic prescribing patterns by indication at the CHUZ/AS in 2022

Antibiotic name	Antibiotic prescription frequency by indication (*N*)			
	Community infections	Medical prophylaxis	Surgical prophylaxis	Total
Ampicillin	18	13	14	45
Metronidazole	9	4	31	44
Ceftriaxone	11	1	21	33
Gentamicin	13	9	5	27
Ciprofloxacin	11	2	9	22
Amikacin	14	4	1	19
Cefotaxime	11	1	0	12
Amoxicillin/clavulanic acid	5	1	3	9
Ofloxacin	2	0	0	2
Ceftriaxone/sulbactam	1	0	1	2
Cefixime	1	0	1	2
Amoxicillin	0	0	1	1
Erythromycin	1	0	0	1
Trimethoprim/sulfamethoxazole	1	0	0	1
Lincomycin	0	0	1	1
Imipenem/cilastatin	1	0	0	1
Azithromycin	0	1	0	1
Total *n* (%)	99 (44.4)	36 (16.1)	88 (39.5)	223

### Distribution of antibiotics according to WHO AWaRe classification at the CHUZ/AS in 2022

**Figure 2.**
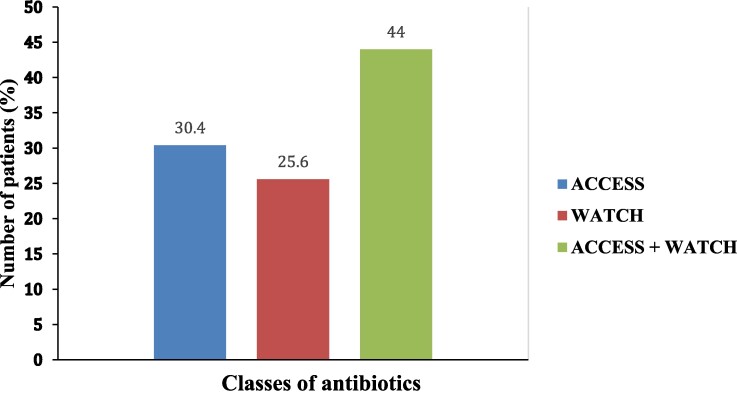
Distribution of antibiotics according to WHO AWaRe classification at the CHUZ/AS in 2022.

The prescribing frequencies for antibiotics classified as Access, Watch or Access + Watch were 30.4%, 25.6% and 44%, respectively. No Reserve antibiotics were prescribed (Figure 2). More than 50% of Access group antibiotics were prescribed in the neonatal and maternity wards. On the other hand, prescribing of Watch group antibiotics alone was relatively low (˂20%) in all departments except surgery and the emergency department. Simultaneous prescribing of the Access and Watch groups was more than 50% in surgery, paediatrics and medicine (Figure 2).

### Antibiotic prescribing practices based on microbiological examination at the CHUZ/AS in 2022

Only 22.7% of inpatients had their specimen processed for microbiological examination/culture and sensitivity testing with only 5.5% treated based on the microbiological outcomes. Therefore, antibiotic therapy was empirical in 94.5% of encounters (Table 6).

**Table 6.** Microbiological use in antibiotic prescribing practices at the CHUZ/AS in 2022

Characteristic	Attribute	Frequency	Percentage
Sampling for microbiological examination	None	85	77.3
	Blood	21	19.1
	Urine	2	1.8
	Cerebrospinal fluid	2	1.8
Type of treatment	Directed	5	5.5
	Empirical	86	94.5

### Consistency between prescribed antibiotics and diagnosis and dosage

Overall, 74.7% of antibiotic prescriptions were consistent with diagnosis, and 80.4% were consistent with dosing (Table 7). Consistency between diagnosis and antibiotic treatment was lowest in the emergency department (50%), and appropriateness with dosing was lowest in neonatal department (76.6%). Ciprofloxacin prescribing was the least consistent for both diagnosis (8%) and dosing (28%) (Table 7).

**Table 7.** Consistency of prescribed antibiotics with diagnosis and dosage at the CHUZ/AS in 2022

Molecules	Diagnostic conformity *n* (%)		Dosing compliance *n* (%)	
	Yes	No	Yes	No
Ampicillin	49 (96.0)	2 (4.0)	48 (94.2)	3 (5.8)
Amoxicillin + clavulanic acid	6 (66.6)	3 (33.4)	8 (88.8)	1 (11.2)
Cefixime	3 (60.0)	2 (40.0)	4 (80.0)	1 (20.0)
Cefotaxime	12 (100)	0	11 (91.6)	1 (8.4)
Ceftriaxone	15 (55.6)	12 (44.4)	24 (88.8)	3 (11.2)
Metronidazole	38 (86.4)	6 (13.6)	36 (81.8)	8 (18.2)
Gentamicin	22 (78.6)	6 (21.4)	26 (92.8)	2 (7.2)
Amikacin	18 (100)	0	14 (77.8)	4 (22.2)
Ciprofloxacin	2 (8.0)	23 (92.0)	7 (28.0)	18 (72.0)
Others^a^	3 (50.0)	3 (50.0)	3 (50.0)	3 (50.0)
Total	168 (74.7)	57 (25.3)	181 (80.4)	44 (19.6)

^a^Others: imipenem, azithromycin, erythromycin and trimethoprim + sulfamethoxazole.

### Prevalence and pattern of antibiotic use per department

Table 8 shows the prevalence and patterns of antibiotic prescribing by department. The prevalence of antibiotics prescribing by department was 100% in the surgical department, 96.9% in neonatal, 95.7% in maternity, 70% in medicine, 60% in paediatrics and 40% in the emergency department (Table 8).

**Table 8.** Overview of prevalence and practice of antibiotic prescribing per department at the CHUZ/AS in 2022

Indicators *n* (%)	Surgical	Neonatal	Maternity	Medicine	Paediatrics	Emergency
Prevalence of antibiotic use						
Yes	16 (100)	31 (96.9)	24 (95.7)	7 (70)	12 (60)	2 (40)
No	0 (0)	1 (3.1)	4 (4.3)	3 (30)	8 (40)	3 (60)
Cumulative number of antibiotics per patient						
1	2 (12.5)	5 (16.1)	1 (4.2)	3 (42.9)	4 (33.3)	2 (100)
2	6 (37.5)	10 (32.3)	19 (79.2)	0 (0)	3 (25.0)	0 (0)
3	7 (43.8)	7 (22.6)	3 (12.5)	3 (42.9)	3 (25.0)	0 (0)
4	1 (6.3)	4 (12.9)	0 (0)	1 (14.3)	1 (8.3)	0 (0)
5	0 (0)	5 (16.1)	1 (4.2)	0 (0)	1 (8.3)	0 (0)
WHO AWaRe classification						
Access	1 (6.3)	18 (58.1)	14 (58.3)	2 (28.6)	3 (25)	0 (0)
Watch	6 (37.5)	2 (6.5)	0 (0)	1 (14.3)	1 (8.3)	2 (100)
Access + Watch	9 (56.3)	11 (35.5)	10 (41.7)	4 (57.1)	8 (66.7)	0 (0)
Type of treatment						
Empirical	16 (100)	26 (83.9)	24 (100)	6 (100)	12 (100)	2 (100)
Documented	0 (0)	5 (16.1)	0 (0)	0 (0)	0 (0)	0 (0)
Pharmacological therapeutic analysis						
Diagnostic conformity	23 (63.9)	82 (87.2)	42 (79.2)	7 (53.8)	15 (53.6)	1 (50)
Dosage compliance	29 (80.6)	72 (76.6)	46 (83.0)	13 (100)	23 (82.1)	2 (100)

## Discussion

This study evaluated the use of antibiotics at the CHUZ/AS in Benin.

The prevalence of antibiotic use (82.9%) at the CHUZ/AS was higher than the 75% reported in Pakistan^53^; 70.6% in Botswana^54^; 60.5% in Ghana across three hospitals^55^; 68.8% in Nigeria^35^; 68%,^56^ 55%^57^ and 46%^58^ in three public referral hospitals in Kenya; 62.3% in Tanzania^33^; 59% in Zambia^46^; 53% in Mauritius^59^; 50% among five African countries in a Global-PPS^60^; 32% in Europe^60^; 38% in America^60^; 39% in Asia^60^; 34.4% in Northern Ireland^61^; and 27.1% in Belgium^62^, respectively. Consequently, the prevalence of antibiotic use reported in our study is lower than the 88.2% reported in Eswatini,^63^ 90% in Pakistan^64^ and 97.6% in Nigeria,^65^ respectively. The WHO recommends that antibiotic prescribing for inpatients should be 40% and below.^29,66^ The prevalence of antibiotic use found in our study and many studies cited above is greater than the recommended threshold for inpatients. Therefore, prescribing of antibiotics should be monitored and AMS programmes be instigated to reduce the overuse of these important medicines.^67,68^ The high prescribing of antibiotics found in this study demonstrates the need to establish and strengthen AMS programmes in Benin.

Regarding factors associated with increased antibiotic prescribing, our study shows that hospital ward, age and surgery since admission were associated with increased antibiotic use at the CHUZ/AS. It is well known that prescribers, patients and healthcare facility infrastructure can contribute to inappropriate antibiotic use, and therefore, judicious mitigation is necessary to ensure appropriate use of these agents and prevent AMR.^69^ Surveillance systems to monitor antimicrobial use (AMU) and resistance patterns are needed. Based on our findings, patients admitted to surgical wards were more likely to receive antibiotics (9.8 times) before and after surgery. In a study carried out in Benin, the findings showed that antibiotic use was high in uninfected patients in surgical (27%) and obstetrics/gynaecology (18%) wards.^51^ Newborns were 34.6 times more likely to be on antibiotics than patients in the age group above 50 years. The overuse of antibiotics in paediatrics has been reported in previous studies.^35,53,70–74^ Children’s conditions are unpredictable, and in some cases, the progression of infectious diseases to fatal outcomes is common, and consequently, some clinicians choose to prescribe antibiotics empirically.^33^

Penicillins, cephalosporins and aminoglycosides were the most commonly used antibiotics (˃20%) before surgery. Overall, the most prescribed antibiotics in this survey were ampicillin, metronidazole and ceftriaxone. For CAIs, ampicillin and amikacin were the most prescribed antibiotics, while for surgical prophylaxis, ceftriaxone and metronidazole were the most prescribed antibiotics. For medical prophylaxis, gentamicin and ampicillin were the most prescribed antibiotics. Consistent with other studies, the overuse of ceftriaxone continues to be an issue in many countries and requires urgent intervention to preserve the efficacy of this Watch group antibiotic.^72,74–79^ A study in Uganda also reported inappropriate use of ceftriaxone among inpatients and recommended urgent interventions to promote rational use of all antibiotics.^80^ Our study findings also revealed that most patients had peripheral catheters which demonstrate the potential high use of injectable antibiotics as evidenced by the overuse of ceftriaxone. This study found that most patients who were treated with antibiotics had CAIs. Our findings are consistent with the results from other studies that found a high use of antibiotics to treat CAIs.^70,80^

In this study, we found substantial evidence that antibiotic use may not be optimal at the CHUZ/AS in Benin. This is because of missed antibiotic doses, a lack of culture and sensitivity testing to guide treatment, a relatively high number of antibiotic prescriptions, particularly in the paediatric and surgical populations, and prescribing a broad-spectrum antibiotic such as ceftriaxone without providing justification. Addressing all these areas will be challenging, particularly given the limited resources for stewardship activities. The use of the CDC^81^ and WHO^82^ guidance documents can direct initial AMS activities according to resource availability, including systematic antibiotic review, antibiotic use surveillance and antibiotic susceptibility testing. The overuse of antibiotics for CAIs and surgical prophylaxis contributes to the emergence and spread of AMR. Evidence has shown that postoperative use of antibiotics should be avoided because it does not yield any benefits but increases the risk of AMR development.^83^ Reducing antibiotic use for prophylaxis through strategies such as infection prevention and control practices is recommended.^84,85^

In our study, only 22.7% of patients had their samples processed for microbiological examination and antibiotics were prescribed based on culture and sensitivity testing in 5.5% of the patients. Consequently, 94.5% of the patients received antibiotics empirically. The lack of both locally generated antibiograms and the use of national clinical guidelines could contribute to suboptimal clinical care and worsening AMR rates. Setting up monitoring committees (drugs and therapeutics, infection prevention and control and AMS committees) and clarifying the roles and responsibilities of these committees in optimizing antibiotic use at the CHUZ/AS could help to improve AMS. The high prescription of antibiotics without guidance from culture and sensitivity testing has been reported in many countries, especially in LMICs.^32,54,63,72,86–90^ Additionally, many LMICs lack antibiograms to guide hospital use of antibiotics.^91–95^ These challenges are worsened by the inadequacies in implementation and functionality of AMS programmes to address AMR.^96,97^ Therefore, there is an urgent need to strengthen the monitoring of AMU, implementation of AMS programmes and laboratory capacity to conduct microbiological tests to promote diagnostic stewardship as reported in other countries.^31,91–94,97–100^

The prescribing frequencies for antibiotics classified as Access, Watch or Access + Watch were 30.4%, 25.6% and 44%, respectively. In Tanzania, on the other hand, a total of 98%, 1.8% and 0.3% of prescribed antibiotics were classified as Access, Watch and Reserve, respectively.^33^ Similarly in Uganda, 47.2% of antibiotics were in the Access group, 44.1% in the Watch group and 9% were not classified.^80^ Our study found that no Reserve antibiotics were prescribed for the inpatients during the survey. Our findings corroborate those reported in other studies.^46,55^ Our findings align with the WHO recommendations of not prescribing Reserve antibiotics frequently and reserves these antibiotics for use to manage MDR pathogens. Over 50% of the Access group antibiotics were prescribed in neonatal and maternity wards. On the other hand, prescribing of Watch group antibiotics alone was relatively low (˂20%) in all departments except surgery and emergency. Simultaneous prescribing of the Access and Watch groups was more than 50% in surgery, paediatrics and medicine. The use of Access group antibiotics in paediatrics was reported to be 47% in Ghana and 100% in Singapore.^43^ Interventions could be adapted to use the WHO AWaRe classification of antibiotics to guide antibiotic use in hospitals in low-resource settings.^37,38,55,101^ Given that antibiotic therapy is mainly empiric at the CHUZ/AS, the high number of Watch group antibiotics prescribed could prove problematic. This gap may be solved effectively and inexpensively through trainings and education of healthcare workers regarding the WHO AWaRe classification of antibiotics which promotes an increase in the use of Access group antibiotics. The WHO has provided materials that can be used to train and educate healthcare workers on AMS.^102,103^

We are aware that this study has some limitations. This study was conducted in one hospital; hence, generalization of the findings must be done with caution. The findings of this study cannot be generalized to the rest of the hospitals in Benin. However, there is a need to avoid the overuse of antibiotics for prophylaxis as this can contribute to the emergence and spread of AMR. These findings demonstrate the need to monitor antibiotic prescribing through instigating and strengthening AMS programmes in Benin. Our findings demonstrate the need to instigate and strengthen AMS programmes in Benin, similar to what other countries are doing.^27,96,97,100,104–108^

## Conclusions

The present study found a high prevalence of antibiotic use among hospitalized patients at the CHUZ/AS in Benin. Additionally, this study found that the hospital was not meeting targets regarding the WHO AWaRe classification of antibiotics. This study provides new information on antibiotic use in a regional hospital and can be used by healthcare institutions and the Ministry of Health to implement or improve AMS programmes, policies and guidelines. It also highlights relevant facts that can be used by other LMICs. Future research should focus on exploring the underlying reasons for antibiotic use by specific antibiotic names or classes, assessing the appropriateness of antibiotic prescribing and identifying the determinants of the effectiveness of AMS programmes, drug and therapeutics committees and infection prevention and control strategies.

## Transparency declarations

The authors have no relevant conflicts of interest to declare. All the authors do not have any financial interests or connections that may directly or indirectly raise concerns of bias in the work reported or the conclusions, implications or opinions made in this publication. The authors declared no potential conflicts of interest concerning the research, authorship and/or publication of this article. The authors declare that the research was conducted in the absence of any commercial or financial relationships that could be construed as a potential conflict of interest.
